# Effect of bone morphogenetic protein-4 on in vitro growth, steroidogenesis and subsequent developmental competence of the oocyte-granulosa cell complex derived from bovine early antral follicles

**DOI:** 10.1186/s12958-016-0137-1

**Published:** 2016-01-15

**Authors:** Yinghua Yang, Chihiro Kanno, Weiping Huang, Sung-Sik Kang, Yojiro Yanagawa, Masashi Nagano

**Affiliations:** Laboratory of Theriogenology, Department of Veterinary Clinical Sciences, Graduate School of Veterinary Medicine, Hokkaido University, Sapporo, 060-0818 Japan

**Keywords:** BMP-4, Developmental competence, Early antral follicle, In vitro growth, Progesterone

## Abstract

**Background:**

Bone morphogenetic proteins (BMPs) play important regulatory roles during folliculogenesis. Theca-derived BMP-4 may be beneficial to in vitro growth culture of early antral follicle-derived oocyte-granulosa cell complexes (OGCs), which is lacking in theca-derived products.

**Methods:**

BMP-4 (0 [control], 10 and 50 ng/mL) was added to growth culture medium. Growth, steroidogenesis and the subsequent developmental competence of OGCs derived from bovine early antral follicles (0.5-1 mm) were examined.

**Results:**

At 4, 8 and 12 days of growth culture, progesterone production by granulosa cells was suppressed by the addition of BMP-4 compared to the control (*P* < 0.05). At 12 days, both the OGC survivability and granulosa cell number in the 50 ng/mL BMP-4 treated group were lower than those of control (48.2 % vs. 67.8 %; 4.96 × 10^4^ vs. 8.5 × 10^4^ cells; *P* < 0.05, respectively), while no difference was found between 10 ng/mL and the control. The mean diameters of granulosa cell in the BMP-4 treated groups were smaller than that of the control (*P* < 0.05). However, the granulosa cell viability, oocyte diameter, oocyte nuclear maturation rate and normal fertilization rate were similar in all of the experimental groups, regardless of the amount of BMP-4 addition (P ˃ 0.05). BMP-4 treated in vitro-grown oocytes showed lower blastocyst rates than untreated ones (*P* < 0.05).

**Conclusions:**

BMP-4 addition during in vitro growth culture suppressed progesterone production and decreased the diameter of granulosa cells, suggesting its effect on steroidogenesis; importantly, it did not affect oocyte growth, nuclear maturation and fertilization. However, BMP-4 impaired subsequent embryonic development, and in higher concentration (50 ng/mL) even compromised OGC viability by suppressing proliferation of granulosa cells.

## Background

Fully grown oocytes in antral follicles (more than 2 mm in diameter) are an important source of in vitro embryo production in cattle [1, 2]. However, the majority of oocytes in an ovary are small oocytes that are either dormant or at various growing stages. It is, therefore, necessary to utilize these small oocytes to make better use of ovaries, especially for species for which ovary samples are extremely rare. Although complete in vitro development of oocytes from primordial follicles has been demonstrated in mice [3, 4], it has not been achieved in other mammals. Among follicles in developmental stages, early antral follicles show the potential to be a supplemental source of oocytes because they are most similar in size to late antral follicles. Several research groups have successfully produced live calves from oocytes released from early antral follicles (less than 1 mm in diameter) after in vitro growth (IVG), in vitro maturation (IVM), in vitro fertilization (IVF) and in vitro culture (IVC) [5–7]. Compared to oocytes grown in vivo, however, the meiotic and developmental competence of IVG oocytes are generally lower [5, 6, 8]. Therefore, it is necessary to improve the IVG culture system.

Theca cells are an essential component of growing follicles, supporting follicle growth and development not only by delivering nutrients and providing the androgens required for conversion into estrogens by granulosa cells (GCs) but also by producing growth factors that can promote follicular development [9]. Bone morphogenetic proteins (BMPs) are members of the transforming growth factor-β superfamily of extracellular signaling molecules, which play multiple roles in the regulation of the growth, differentiation and apoptosis of numerous cell types. Theca-derived BMP-4 has been shown to be capable of regulating the growth and function of GCs by suppressing apoptosis and enhancing the secretion of estradiol, while reducing progesterone (P_4_) secretion in vitro, an action consistent with a delay of luteinization and/or atresia [10, 11]. Furthermore, previous in vivo studies have shown that BMP-4 mRNA was expressed at high levels in the theca of developing dominant follicles, while it was very low or undetectable in atretic follicles [12]. This evidence suggest the possibility that theca-derived BMP-4 is related to the emergence and/or maintenance of dominant follicles; thus, it may contribute to IVG, which is aimed at producing healthy oocytes similar to those grown in the dominant follicles. However, the effects of BMP-4 on oocyte growth and subsequent developmental competence are unknown because GCs alone were studies in previous studies [10, 13, 14].

The current IVG protocol for bovine oocytes only uses theca-free oocyte-granulosa cell complexes (OGCs). As a result, the products from theca cells, especially growth factors, such as BMP-4, are absent. Furthermore, a previous study showed P_4_ concentration (68 ng/ml) in the follicular fluid of the growing antral follicles with a low antral follicle count (AFC) were double that in follicles with a high AFC (32 ng/ml) [15]. It was also reported that oocytes derived from follicles with a low AFC had a much lower blastocyst formation rate [15]. A low AFC is associated with diminished ovarian function and fertility in both human and cow [16, 17]. Our preliminary study found P_4_ concentration in the IVG culture medium soared above 60 ng/ml, a high level comparable to that in follicles with a low AFC. Therefore, in an attempt to compensate for the missing roles of theca cells and reduce P_4_ production during IVG culture, the aim of our study was clarifying the functions of BMP-4 produced by theca cells. We asked whether BMP-4 adding in the IVG culture medium affects OGC growth, P_4_ production and subsequent developmental competence acquisition in the present study.

## Methods

### Collection of OGCs and IVG culture

Bovine ovaries obtained from a slaughterhouse were kept in plastic bags at 20 °C and were transported to the laboratory within 6 to 10 h after collection. After three washes in saline, sliced ovarian cortex strips (<1 mm thick) were prepared using a surgical blade (No. 11). Early antral follicles (0.5-1 mm in diameter) were dissected from cortex strips using a surgical blade (No. 20) under a stereomicroscope in TCM199 (Invitrogen; Grand Island, NY, USA) supplemented with 0.1 % polyvinyl alcohol, 25 mM HEPES, 10 mM sodium bicarbonate, and 50 μg/mL gentamicin sulfate (isolation medium, pH 7.4), as described elsewhere [18]. Follicles were punctured to release OGCs using a pair of fine forceps, and the OGCs with a normal appearance were individually cultured for 12 days in 96-well culture plates (Becton, Dickinson and Co., Franklin Lakes, NJ, USA) with 200 μL of growth medium at 39 °C in humidified air with 5 % CO_2_. The growth medium was HEPES-buffered TCM199 supplemented with 0.91 mM sodium pyruvate, 1 μg/mL estradiol-17β, 5 % fetal calf serum (FCS; Invitrogen), 4 mM hypoxanthine, 4 % polyvinylpyrrolidone (MW 360,000) and 50 μg/mL gentamicin sulfate. Half of the medium was replaced every 4 days, throughout the culture, 10 and 50 ng/ml BMP-4 (HumanZyme, Inc., Chicago, IL, USA) was added to IVG medium while 0 ng/ml BMP-4 group worked as non-treated control. The doses of BMP-4 were selected based on previous in vitro studies, in which BMP-4 at these doses was demonstrated to be effective in suppressing GC apoptosis and P_4_ production [10, 14].

### P_4_ assay

The culture medium collected at 4, 8 and 12 days of IVG culture was frozen at −30 °C until P_4_ assay by using enzyme immunoassay, as previously described [19]. IVG media were loaded directly as a sample to the well of a microplate. Anti-progesterone-3CMO-BSA (KZ-HS-P13, Cosmo Bio Co., Ltd., Tokyo, Japan) was used as the primary antibody, and goat anti-rabbit serum (111-005-003, Jackson Immuno Research Laboratories, Inc., PA, USA) was used as the secondary antibody. The intra- and inter-assay coefficients of variations were 5.8 % and 9.8 %, respectively.

### Evaluation of OGC growth

In the present study, the viability of OGC, as well as the diameter, viability and number of GCs, were considered as OGC growth parameters. Before and after IVG culture, OGC growth parameters were measured. The survivability of OGCs was evaluated by their morphological appearance; oocytes completely enclosed by several healthy granulosa cell layers were considered to be viable [18]. The diameter, viability and number of GCs from viable OGCs were assessed using an acridine orange/propidium iodide cell viability kit together with a cell counter (F23001 and L2000, respectively; Logos Biosystems, Gyunggi, Republic of Korea). To prepare dispersive GCs for the counting, culture medium in the well of each viable OGC was removed and replaced with 100 μL of D-PBS (−) supplemented with 0.125 % trypsin and 0.05 % EDTA. After 10 min of trypsinization and pipetting several times, 25 μL of FCS was added to stop the digestion, and then, the denuded oocyte was removed from the well and discarded.

### IVM of in vivo-grown and IVG oocytes

In vivo-derived oocytes, serving as control, were collected from antral follicles (2–8 mm diameter) and submitted to IVM as described previously [20]. Briefly, cumulus-oocyte complexes (COCs) were incubated in droplets of IVM medium (approximately 10 COCs/50 μL) and were then covered with paraffin oil for 22 h at 39 °C in a humidified atmosphere with 5 % CO_2_. The maturation medium consisted of HEPES-buffered TCM199 supplemented with 0.2 mM sodium pyruvate, 0.02 units/mL FSH (from porcine pituitary), 1 μg/mL estradiol-17β, 10 % FCS, and 50 mg/mL gentamicin sulfate.

For IVG oocytes, COCs were isolated from the viable OGCs for pre-IVM and IVM as previously described [7]. Briefly, oocytes were individually cultured in individual wells of micro-well plates (MiniTrays 163118; NUNC, Roskilde, Denmark) that contained 6 mL of pre-IVM medium, and then, 6 mL of IVM medium for 10 and 22 h at 39 °C in a humidified atmosphere with 5 % CO_2_ [21]. Pre-IVM medium, which is modified from IVM medium, contained additional 0.5 mM 3-isobutyl-1-methylxanthine, a phosphodiesterase inhibitor, and a lower FSH concentration (2 × 10^−6^ units/mL) [7].

Before IVG and after IVM culture, all 92 oocytes were measured for their diameters as previously described [18]. Briefly, the diameter of each denuded oocyte was measured using an inverted microscope (CK40, Olympus, Tokyo, Japan) connected to a CCD camera (Moticam 2000, Shimadzu Rika Corporation, Tokyo, Japan) and image processing software (Motic Images Plus 2.2 s, Shimadzu).

### IVF and IVC

IVF was performed according to a previously described procedure [22]. Briefly, COCs were co-incubated with frozen-thawed motile sperm (5 × 10^6^ sperm/mL) separated by a Percoll gradient (45 % and 90 %). Approximately 10 COCs were cultured in a 100-μL droplet of modified Brackett and Oliphant isotonic medium containing 3 mg/mL fatty acid–free BSA and 2.5 mM theophylline for 18 h at 39 °C in a humidified atmosphere with 5 % CO_2_, 5 % O_2_ and 90 % N_2_.

Only 10 ng/mL BMP-4 treatment were investigated for IVC because a low survival rate in 50 ng/mL BMP-4 treated OGCs was observed during IVG. IVC was performed as previously described [20]. Briefly, presumptive zygotes were denuded by vortexing and washed three times in culture medium. Then approximately 30 zygotes were cultured in a 30-μL droplet of culture medium for 6 days at 39 °C in a humidified atmosphere with 5 % CO_2_, 5 % O_2_ and 90 % N_2_. The culture medium consisted of modified synthetic oviduct fluid containing 1 mM glutamine, twelve essential amino acids for basal medium Eagle, seven nonessential amino acids for minimum essential medium, 10 μg/mL insulin, and 5 mM glycine, 5 mM taurine, 1 mM glucose, and 3 mg/mL fatty acid-free BSA.

### Evaluation of oocyte nuclear maturation and fertilization

Following IVM or IVF, oocytes were denuded from cumulus cells by vortexing and were then stained with 1 % aceto-orcein. To evaluate nuclear maturation, the nuclear status was classified as germinal vesicle (GV), metaphase I (MI), anaphase I/telophase I (AI/TI), and metaphase II (MII) by observation under a phase-contrast microscope. The diameter of each denuded oocyte after IVM (before fixation) was measured and was considered the oocyte diameter after IVG culture. To evaluate fertilization, the oocytes were considered to be penetrated by sperm when they had one or more enlarged sperm head (s) or male pronucleus (ei) with corresponding sperm tail (s). Monospermic penetration was defined as normal fertilization in which oocytes have a single male pronucleus and a female pronucleus, or a single enlarged sperm head with the chromosomes at AII/TII. Cleavage and the blastocyst rates were determined after 2 days and 6 days of IVC, respectively, and the cell numbers in blastocysts were counted using an air-drying method [23].

### Statistical analysis

All statistical analyses were performed using JMP software version 11.0.0 (SAS Institute, Cary, NC, USA). The effects of the BMP-4 treatments on the GC diameter, viability and number, oocyte diameter and blastocyst cell number were analyzed by one-way ANOVA followed by Turkey-Kramer’s HSD as a post hoc test. The effects of the BMP-4 treatments and culture duration on the P_4_ concentration were analyzed by two-way ANOVA followed by Turkey-Kramer’s HSD as a post hoc test. The effect of the BMP-4 treatments on the OGC viability, oocyte nuclear maturation and fertilization, and blastocyst rate were analyzed by a chi-square test.

## Results

### Growth of OGC

Table 1 shows the OGC growth parameters before and after IVG culture with or without BMP-4 treatments. After 12 days of IVG culture, 50 ng/mL BMP-4 treated OGCs showed lower viability (48.2 %, *P* < 0.05) compared to others (~70 %). The viability, diameter and number of GCs significantly increased (*P* < 0.05) compared to those of the pre-IVG culture. The GC viabilities in all of the experimental groups were similar; however, the GC number in the 50 ng/mL BMP-4 treated group was lower than that of the control group (*P* < 0.05). In both BMP-4 treatments, a smaller mean diameter of GCs (11.2 and 11.0 μm) was observed compared to that of the controls (12.0 μm) (*P* < 0.05).

**Table 1:** Effect of BMP-4 in IVG medium on the growth of OGCs from early antral follicles

IVG culture (day)	BMP-4 (ng/ml)	% of OGC viability (n)	Granulosa cell		
			Counting (× 10^3^ cells)	% of viability (n)	Diameter (μm)
0	-	100.0^a^ (30)	2.0 ± 0.5^c^	88.7 ± 2.8^b^ (30)	9.3 ± 1.1^c^
12	0	67.8^b^ (242)	85.0 ± 33.7^a^	92.8 ± 3.4^a^ (26)	12.0 ± 0.8^a^
	10	70.4^b^ (250)	69.0 ± 23.5^ab^	93.2 ± 2.9^a^ (25)	11.2 ± 0.9^b^
	50	48.2^c^ (247)	49.6 ± 12.4^b^	93.7 ± 3.2^a^ (14)	11.0 ± 0.9^b^

Data are presented as the mean ± standard deviation

^abc^Values with different superscripts in the same column are significantly different (*p* < 0.05)

### P4 production of OGC

**Fig. 1:**
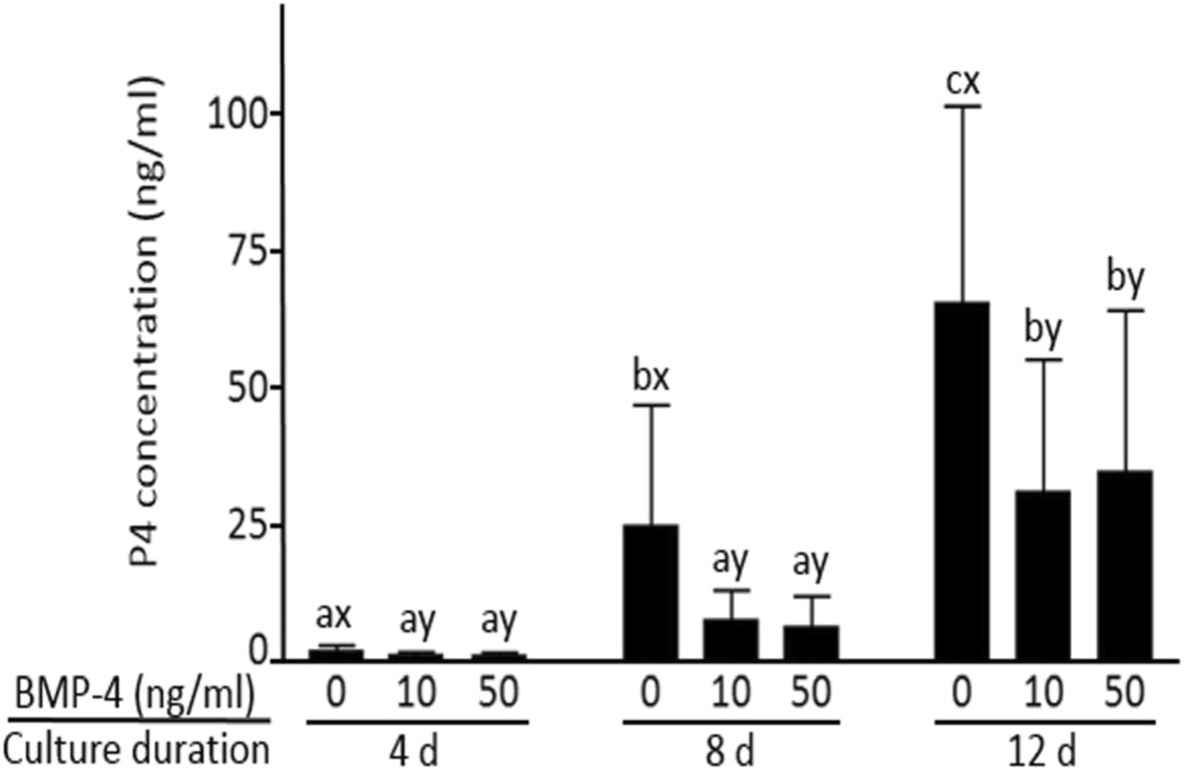
Effect of BMP-4 on progesterone (P_4_) production by GCs during IVG culture. ^abc^Different letters represent differences among the same doses of BMP-4 (*P* < 0.05). ^xy^Different letters represent differences within same day of culture (*P* < 0.05). Bars represent the standard deviation

As shown in Fig. 1, the P_4_ concentration in the culture medium of the control group increased throughout the duration of IVG culture (*P* < 0.05). However, in the BMP-4 treated groups, the P_4_ concentration was decreased on day 8 and 12 of IVG culture in comparison to control (*P* < 0.05). There was no difference in P_4_ production between BMP-4 treated groups.

### Subsequent developmental competence

As shown in Tables 2 and 3, the oocyte diameter, nuclear maturation and fertilization were not significantly affected by the BMP-4 treatments, although there was a decreasing tendency of total penetration in the 50 ng/mL BMP-4 treated group. IVG oocytes, regardless of BMP-4 addition, were smaller and had lower rates of normal fertilization and total penetration than in vitro-grown oocytes (*P* < 0.05). As shown in Table 4, the cleavage rates after IVF were similar between BMP-4 treated and non-treated IVG oocytes; however, BMP-4 treated oocytes had lower blastocyst rates, based on both inseminated oocytes and cleaved oocytes (*P* < 0.05). Compared to the in vivo-grown oocytes, IVG oocytes showed lower cleavage and blastocyst rates (*P* < 0.05).

**Table 2:** Effect of BMP-4 in IVG medium on the nuclear maturation and diameter of IVG oocytes

Oocyte	BMP-4 (ng/ml)	No. of oocytes cultured (replicates)	Mean oocyte diameter (μm)		% of nuclear status (no.)		
			Before IVG	After IVM	M I	A I/ T I	M II
In vitro grown	0	33^c^ (4)	98.9 ± 3.4	116.9 ± 4.1^b^	6.5 (2)	0	93.5 (29)
	10	29 (4)	99.4 ± 3.0	117.7 ± 3.5^b^	10.3 (3)	0	89.7 (26)
	50	30 (4)	99.1 ± 3.1	116.8 ± 2.9^b^	3.3 (1)	3.3 (1)	93.4 (28)
In vivo grown^d^	-	32 (3)	-	120.2 ± 3.1^a^	6.3 (2)	0	93.8 (30)

Abbreviations: M I metaphase I, A I/T I anaphase I/telophase I, M II metaphase II

^ab^Values with different superscripts in the same column are significantly different (*p* < 0.05)

^c^For the evaluation of nuclear maturation, 31 oocytes in the 0 ng/ml BMP-4 treated group were used because 2 oocytes were lost during fixation

^d^Oocytes collected from antral follicles (2–8 mm in diameter) served as in vivo-derived controls

**Table 3:** Effect BMP-4 in IVG medium on the fertilization of IVG oocytes

Oocyte	BMP-4 (ng/ml)	No. of oocytes (replicates)	% of normal fertilization			% of polyspermy	% of total penetration
			ESH	2PN	subtotal		
In vitro grown	0	73 (9)	16.4	43.8^b^	60.2^b^	6.8	67.0^b^
	10	91 (9)	13.2	48.4^b^	61.6^b^	9.9	71.5^b^
	50	53 (9)	5.7	41.5^b^	47.2^b^	7.5	54.7^b^
In vivo grown^c^	-	83 (3)	6.0	80.7^a^	86.7^a^	7.2	93.9^a^

Abbreviations: ESH enlarged sperm head, 2PN two pronuclei

^ab^Values with different superscripts in the same column are significantly different (*p* < 0.05)

^c^Oocytes collected from antral follicles (2–8 mm in diameter) served as in vivo-derived controls

**Table 4:** Effect of BMP-4 in IVG medium on the development of IVG oocytes after IVF

Oocyte	BMP-4 (ng/ml)	No. of oocytes (replicates)	% of cleaved oocytes	% of blastocysts based on		Cell no. in blastocysts (n)
				Inseminated oocytes	Cleaved oocytes	
In vitro grown	0	257 (8)	54.9^b^	9.3^b^	17.0^b^	91.4 ± 13.1^b^ (24)
	10	239 (7)	47.3^b^	1.3^c^	2.7^c^	142.0 ± 45.6^d^ (2)
In vivo grown^e^	-	150 (5)	76.7^a^	25.3^a^	33.0^a^	164.6 ± 10.4^a^ (19)

^abc^Values with different superscripts in the same column are significantly different (*p* < 0.05)

^d^One blastocyst was lost during cell counting; as a result, no statistical comparison was conducted

^e^Oocytes collected from antral follicles (2–8 mm in diameter) served as in vivo-derived controls

## Discussion

In the current study, BMP-4 suppressed P_4_ production from GCs, in agreement with the results of other in vitro studies [13, 14]. It has been reported that the inhibition of the acetylation of histone H3 associated with the steroidogenic acute regulatory protein promoter region by BMP-4 may be one of the underlying molecular mechanisms of the inhibition of P_4_ synthesis in GCs [13]. Because increased P_4_ synthesis is a characteristic of the GC luteinization process [24], BMP-4 showed an effect of anti-luteinization. This effect was further verified by the significantly smaller diameter (~11 μm) of GCs in the BMP-4 treated groups after IVG culture compared to non-BMP-4 treated control (12.0 μm). It has been reported that both in vivo-grown large luteal cells originated from GCs and in vitro-luteinized GCs have similar sizes, which are larger than that of GCs in pre-ovulatory follicles (mean diameter, 38.4 vs.10.6 μm, respectively) [25, 26]. Therefore, the delay in GC enlargement observed in the present study may be attributed to delayed luteinization, which is associated with reduced P_4_ synthesis in GCs treated with BMP-4. It is known that GCs become luteinized and P_4_ secretion from GCs increases in atretic follicles [27, 28]. In the present study, however, BMP-4 treatment reduced P_4_ to a level (~33 ng/ml) close to that of follicular fluid (~32 ng/ml) in growing antral follicles with high AFC [17]. These data indicate the possibility for developing an in vitro model mimicking the growth of bovine oocytes in healthy follicles.

Despite the similarity in the ability to delay luteinization, the 50 ng/mL BMP-4 treatment caused a decrease in the GC number relative to the controls, while 10 ng/mL BMP-4 did not. It appears that BMP-4 impaired proliferation, but did not induce apoptosis of GCs, as the viability of GCs was high (more than 90 %) in all of the experimental groups. This finding is inconsistent with previous studies in which 50 or 100 ng/mL BMP-4 did not affect GC proliferation over 6 or 4 days of GC culture, respectively [13, 14]. We didn’t expected the GC number to decrease with BMP-4 treatment because it has been demonstrated that BMP-4 could reduce apoptosis of GCs by suppressing the action of caspase-activated DNase induced by Survivin, a member of the inhibitor of apoptosis family [10]. One major difference between the present and the previous studies is that we cultured OGCs, rather than GCs alone. The involvement of oocytes complicates the culture system and may be responsible for the discrepancies. It is now widely recognized that oocyte secreted factors (OSFs), such as growth differentiation factor-9 (GDF-9) and BMP-15, direct the functions of their surrounding GCs, including the promotion of cell growth and prevention of cell death and luteinization [29–32]. Because the types of receptors for the transforming growth factor-β superfamily are limited, ligands of the BMP/GDF subfamily bind to receptors in a shared manner [33]. Among the limited receptors, BMP type-II receptor is the sole type-II receptor presenting in GCs for GDF-9 and BMP-15 and is also an important receptor for BMP-4 [34]. In follicles, GDF-9 and BMP-4 could induce GCs to produce Gremlin, which is known to be effective at antagonizing BMP-4 actions without affecting oocyte-derived GDF-9 and BMP-15 [31, 35, 36]. As a result, it is proposed that oocyte could maintain its surrounding microenvironment, which is important for oocyte development, from the actions of theca-derived BMPs [31]. Take into consideration of the fact that a 20-fold excess of gremlin to BMP-4 was needed to completely block BMP-4 action [35], exogenous BMP-4 at the doses studied (10 and 50 ng/ml) may be too high and probably impaired the function of OSFs by competitively binding receptors against OSFs, resulting in decreased GC proliferation in a dose-dependent manner. A smaller number of GCs in the 50 ng/mL BMP-4 treated group, in turn, may lead to the decreased OGC viability when consider the fact that oocytes also relay on the support of GCs for long-term growth.

In the present study, BMP-4 treatment did not promote oocyte growth. However, BMP-7, another theca-derived growth factor, increased the oocyte diameter and volume during IVG, in which OGCs were cultured in groups on membrane inserts [37]. The culture system may partially cause the different results, but unknown differences in the biological function between BMP-4 and–7 may also be responsible [34]. Our study showed that BMP-4 impaired embryo development of IVG oocytes without affecting oocyte nuclear maturation and fertilization, indicating that BMP-4 may inhibit the cytoplasmic maturation of oocytes. Another study reported that BMP-4 addition during IVM of COC had no effect on bovine oocyte nuclear maturation and subsequent embryo development [38]. The differences in the growth stage of oocytes and exposure time to BMP-4 may have led to the discrepancy. Through OSFs, oocytes appear to control their neighboring somatic cells, directing them to perform functions required for the appropriate development of oocytes. The presence of this regulatory loop was demonstrated by the fact that the neutralization of OSFs by antagonists of BMP-15 and GDF-9 during IVM impaired the developmental competence of COCs [39]. Although there was no BMP-4 addition during pre-IVM and IVM in the present study, the presumed interruption of the regulatory loop by BMP-4 during IVG appeared to cause a lasting adverse effect, as shown in the subsequent developmental competence of oocytes. Further investigations are necessary to verify this type of receptor competition between BMP-4 and OSFs and its consequential effects.

Compared to in vivo-grown oocytes, IVG oocytes showed inferior competences for fertilization and development to blastocyst, although their nuclear maturation was similar to in vivo-grown ones, indicating that the cytoplasmic maturation of IVG oocytes might be inadequate.

To our knowledge, this is the first study utilizing OGCs model to investigate the BMPs on growth, steroidogenesis and subsequent developmental competence of OGCs derived from bovine early antral follicles. This model will be helpful for studying the function of BMPs or other growth factors in growing antral follicles in a more comprehensive way.

## Conclusions

BMP-4 addition during IVG culture suppressed P_4_ production from GCs and decreased the diameter of GC, suggesting its effect on steroidogenesis; importantly, it did not affect oocyte growth, nuclear maturation and fertilization. However, BMP-4 impaired embryonic development maybe due to insufficient cytoplasmic maturation and in higher concentration (50 ng/mL) even compromised OGC viability by suppressing GC proliferation. In future studies, we should investigate the cytoplasmic maturation of IVG oocytes treated with BMP-4; moreover, we should develop the IVG system for the production of oocytes with high developmental competence without GC luteinization.

## Abbreviations

2PN: Two pronuclei
AFC: Antral follicle count
AI/TI: Anaphase I/telophase I
ANOVA: analysis of variance
BMPs: Bone morphogenetic proteins
COC: Cumulus-oocyte complex
DNase: Deoxyribonuclease
D-PBS: Dulbecco’s phosphate buffered saline
EDTA: Ethylenediaminetetraacetic acid
ESH: Enlarged sperm head
FCS: Fetal calf serum
FSH: Follicle-stimulating hormone
GC: Granulosa cell
GDF: Growth differentiation factor
GV: Germinal vesicle
HEPES: 2-[4-(2-Hydroxyethyl)-1-piperazinyl] ethanesulfonic Acid
HSD: Honestly significant difference
IVC: in vitro culture
IVF: in vitro fertilization
IVG: in vitro growth
IVM: in vitro maturation
MI: metaphase I
MII: metaphase II
mRNA: messenger RNA
OGC: oocyte-granulosa cell complex
OSF: oocyte secreted factor
P_4_: progesterone
TCM199: tissue culture medium 199
